# CD134 as target for specific drug delivery to auto-aggressive CD4^+ ^T cells in adjuvant arthritis

**DOI:** 10.1186/ar1722

**Published:** 2005-03-21

**Authors:** Elmieke PJ Boot, Gerben A Koning, Gert Storm, Josée PA Wagenaar-Hilbers, Willem van Eden, Linda A Everse, Marca HM Wauben

**Affiliations:** 1 Department of Pharmaceutics, Utrecht Institute for Pharmaceutical Sciences, Utrecht University, Utrecht, The Netherlands; 2 Division of Immunology, Department of Infectious Diseases and Immunology, Faculty of Veterinary Medicine, Utrecht University, Utrecht, The Netherlands

## Abstract

T cells have an important role during the development of autoimmune diseases. In adjuvant arthritis, a model for rheumatoid arthritis, we found that the percentage of CD4^+ ^T cells expressing the activation marker CD134 (OX40 antigen) was elevated before disease onset. Moreover, these CD134^+ ^T cells showed a specific proliferative response to the disease-associated epitope of mycobacterial heat shock protein 60, indicating that this subset contains auto-aggressive T cells. We studied the usefulness of CD134 as a molecular target for immune intervention in arthritis by using liposomes coated with a CD134-directed monoclonal antibody as a drug targeting system. Injection of anti-CD134 liposomes subcutaneously in the hind paws of pre-arthritic rats resulted in targeting of the majority of CD4^+^CD134^+ ^T cells in the popliteal lymph nodes. Furthermore, we showed that anti-CD134 liposomes bound to activated T cells were not internalized. However, drug delivery by these liposomes could be established by loading anti-CD134 liposomes with the dipalmitate-derivatized cytostatic agent 5'-fluorodeoxyuridine. These liposomes specifically inhibited the proliferation of activated CD134^+ ^T cells *in vitro*, and treatment with anti-CD134 liposomes containing 5'-fluorodeoxyuridine resulted in the amelioration of adjuvant arthritis. Thus, CD134 can be used as a marker for auto-aggressive CD4^+ ^T cells early in arthritis, and specific liposomal targeting of drugs to these cells via CD134 can be employed to downregulate disease development.

## Introduction

In several autoimmune diseases, for example rheumatoid arthritis, the involvement of CD4^+ ^T cells in disease induction has been suggested [1]. As a treatment strategy, the manipulation of CD4^+ ^T cells by CD4-directed antibodies has therefore been studied extensively [2]. However, because anti-CD4 therapy targets the whole CD4^+ ^population, CD4^+ ^T cells not related to the disease or involved in disease regulation will also be affected. Ideally, only the auto-aggressive CD4^+ ^T cells that are involved in the disease process should be targeted. Because for many human autoimmune diseases the exact antigens recognized by these cells are not known, a therapy would be favorable that specifically targets the auto-aggressive CD4^+ ^T cells and does not depend on the definition of the crucial auto-antigen.

Because auto-reactive CD4^+ ^T cells become activated upon recognition of their cognate antigen in the periphery, they will be transiently marked by the expression of T cell activation markers. In this respect, CD134 (OX40 antigen) is an interesting candidate target molecule, because CD134 is expressed *in vivo *exclusively on activated CD4^+ ^T cells (reviewed in [3]). In experimental autoimmune encephalomyelitis, a disease model for multiple sclerosis, it has been shown that CD134 is preferentially expressed on pathogenic CD4^+ ^T cells that home to the target organ (namely the central nervous system) [4], and transiently marks the auto-aggressive T cells specific for myelin basic protein [5]. Moreover, in this T cell transfer model, depletion of CD134^+ ^T cells with an anti-CD134 immunotoxin results in the amelioration of paralytic symptoms [6]. Interestingly, in patients with rheumatoid arthritis a high percentage of CD4^+ ^T cells in synovial fluid express CD134 in comparison with peripheral blood T cells [6,7], suggesting that auto-aggressive CD4^+ ^T cells may be transiently marked by surface expression of CD134 in arthritis too.

Here, we investigated whether CD134 can be used as a target for specific drug delivery to activated auto-aggressive CD4^+ ^T cells in arthritis. For this purpose, the rat adjuvant arthritis (AA) model was studied. In this model, a syndrome resembling rheumatoid arthritis is actively induced in Lewis rats after immunization with *Mycobacterium tuberculosis *(Mt) in adjuvant [8]. We first analyzed the CD134 expression on CD4^+ ^T cells during AA, and investigated the presence of auto-aggressive T cells within the CD134^+^CD4^+ ^T cell subset. We also studied drug delivery to CD134^+ ^T cells both *in vitro *and *in vivo *using liposomes coated with a CD134-directed monoclonal antibody (mAb) as a drug targeting system. To investigate the possibility for therapeutic intervention in arthritis, anti-CD134 liposomes were loaded with a cytostatic drug and administered early in actively induced arthritis. We show that CD134 can be used as a marker for activated auto-aggressive T cells early in AA, that targeting of these cells *in vivo *can be achieved with anti-CD134 liposomes, and that the course of AA could be affected with drug-containing anti-CD134 liposomes.

## Materials and methods

### Animals

Male inbred Lewis rats were obtained from the University of Limburg (Maastricht, The Netherlands) and were used between 7 and 10 weeks of age. The animals were kept under conventional conditions and had access to standard pelleted rat chow and acidified water *ad libitum*. The Utrecht University Animal Ethics Committee approved all animal experiments.

### Antigens

Heat-killed Mt, strain H37RA, was obtained from Difco Laboratories (Detroit, Michigan, USA). For immunization, Mt was suspended in incomplete Freund's adjuvant (Difco Laboratories). Peptides Mt HSP60_176–190 _(EESNTFGLQLELTEG; one-letter amino acid codes) (HSP60 stands for heat shock protein 60), Mt HSP60_211–225 _(AVLEDPYILLVSSKV) and OVA_323–339 _(ISQAVHAAHAEINEAGR) (OVA stands for Ovalbumin) were obtained from Isogen Bioscience (Maarssen, The Netherlands).

### mAbs and second-step reagents

The anti-CD134 (OX40) and anti-CD25 (OX39) hybridomas were obtained from the ECACC (Salisbury, UK) [9]. The 12CA5 hybridoma producing IgG2b isotype control mAb was kindly provided by Dr GJ Strous (Department of Cell Biology and Institute of Biomembranes, University Medical Center, Utrecht, The Netherlands). mAbs were isolated from hybridoma supernatant by affinity chromatography with GammaBind Plus Sepharose (Roche Pharmacia, Uppsala, Sweden). For ease of flow cytometric detection, some purified mAbs were biotinylated with D-biotinoyl-ε-aminohexanoic acid-*N*-hydroxy-succinimide ester (Roche Molecular Biochemicals, Basel, Switzerland). Fluorescein isothiocyanate (FITC)-conjugated anti-CD4 (OX35) and anti-CD45RA (OX33), phycoerythrin (PE)-conjugated goat-anti-mouse immunoglobulin, PE-conjugated streptavidin, peridinin chlorophyll protein (PerCP)-conjugated anti-T-cell antigen receptor (anti-TCR)-αβ (R73) and IgG1 isotype control (A112), and allophycocyanin-conjugated streptavidin were purchased from BD Pharmingen (San Diego, California, USA).

### Culture of rat CD4^+ ^T cell clone A2b

The isolation, maintenance, and properties of rat CD4^+ ^T cell clone A2b have been described previously [10]. The arthritogenic T cell clone A2b recognizes the 180 to 188 epitope of *Mycobacterium tuberculosis *HSP60 [11]. Cells were cultured in medium (Iscove's modified Dulbecco's medium (Invitrogen, Merelbeke, Belgium), supplemented with L-glutamine (2 mM), 2-mercaptoethanol (50 μM), penicillin (50 U/ml) and streptomycin (50 μg/ml)) with 2% heat-inactivated normal rat serum.

### Induction of AA

Rats were injected intradermally with 100 μl of Mt in incomplete Freund's adjuvant at the base of the tail. For studying cell-surface marker expression, CD4^+ ^subset specificity during AA and liposome binding *in vivo*, 10 mg/ml Mt was used. For AA treatment studies, rats were immunized with 5 mg/ml Mt (yielding 100% disease incidence, but lower maximum disease scores in comparison with 10 mg/ml Mt). Rats were weighed and examined for clinical signs of arthritis in a semi-blinded set-up. Severity of arthritis was scored by grading each paw from 0 to 4 based on erythema, swelling and immobility of the joints, resulting in a maximum score of 16 per animal [12].

### *Ex vivo *analysis of cell-surface marker expression

Before Mt immunization or 7, 10, 14, 21 or 35 days afterwards, rats were killed and popliteal lymph nodes (PLN), inguinal lymph nodes (ILN), spleen, and peripheral blood were isolated. Single-cell suspensions were prepared by mechanically forcing the organs through a 70 μm mesh; erythrocytes were removed from the splenocyte and blood suspensions by Ficoll-Isopaque gradient centrifugation. Cells (2 × 10^5 ^per sample) were labeled with anti-CD134 for 30 min on ice, followed by incubation with PE-conjugated goat anti-mouse immunoglobulin and subsequently with anti-CD4-FITC. The cells were incubated and washed (between each labeling step) in blocking buffer (PBS (Cambrex Bio Science, Verviers, Belgium) containing 4% heat-inactivated rat serum, 1% fraction V BSA (Sigma-Aldrich Chemie, Zwijndrecht, The Netherlands) and 0.1% NaN_3_). Finally, cells were washed in PBS, fixed in 2% paraformaldehyde and stored at 4°C in the dark. Cell-associated fluorescence was analyzed within 10 days on a FACSCalibur using Cell Quest software (Becton Dickinson, Brussels, Belgium).

### Cell sorting and *ex vivo *proliferation of CD4^+ ^T cell subsets

At 7 and 10 days after Mt immunization, PLN, ILN, and spleens of 10 to 15 rats were isolated and each organ type was pooled. Single-cell suspensions were prepared as described above. Cells were washed and incubated in PBS containing 4% heat-inactivated rat serum, and stained with anti-CD134-biotin/streptavidin-allophycocyanin and anti-CD4-FITC. CD4^+^, CD4^+^CD134^- ^and CD4^+^CD134^+ ^cells were sorted with a FACS Vantage and Cell Quest software (Becton Dickinson), resulting in fractions that were 87 to 97% pure.

Sorted cells were washed and incubated for 72 hours in medium with 2% heat-inactivated normal rat serum in flat-bottomed 96-well plates (Corning-Costar, Schiphol, The Netherlands) at 5 × 10^4 ^cells per well in the presence of 30 Gy-irradiated thymocytes as antigen-presenting cells (APC) (10^6 ^cells per well) and concanavalin A (Con A; 2.5 μg/ml) or antigen (20 μg/ml Mt HSP60_176–190_, 20 μg/ml Mt HSP60_211–225_). Finally, cells were pulsed for 18 to 20 hours with [^3^H]thymidine, 0.4 μCi per well (specific radioactivity 1 Ci/mmol; Amersham Biosciences, Roosendaal, The Netherlands), after which [^3^H]thymidine incorporation was measured. Results are presented as the mean stimulation index (SI, defined as [^3^H]thymidine incorporation in the presence of antigen or Con A divided by [^3^H]thymidine incorporation in the absence of antigen or Con A) of triplicate wells. For logistic reasons, ILN single-cell suspensions were kept overnight on ice and were washed, stained and sorted on the following day.

### Preparation of (mAb-coupled) PEG-liposomes

Liposomes were composed of egg phosphatidylcholine, cholesterol, poly(ethyleneglycol)_2000_-distearoylphosphatidylethanolamine (PEG_2000_-DSPE) and maleimide-PEG_2000_-DSPE in a molar ratio of 2:1:0.075:0.075. Egg phosphatidylcholine was kindly provided by Lipoid (Ludwigshafen, Germany), PEG_2000_-DSPE was purchased from Avanti Polar Lipids (Birmingham, Alabama, USA), cholesterol from Sigma-Aldrich Chemie, and Maleimide-PEG_2000_-DSPE from Shearwater Polymers (Huntsville, Alabama, USA). Liposomes used for investigating liposome binding *in vivo *contained 0.1 mol% 1,1'-dioctadecyl-3,3,3',3'-tetramethylindodicarbocyanine, 4-chlorobenzenesulfonate salt (DiD; Molecular Probes Europe, Leiden, The Netherlands). Liposomes used for investigating *in vitro *binding and internalization contained 0.1 mol% Texas red-phosphatidylethanolamine (Molecular Probes Europe). Liposomes used for studying drug delivery *in vitro *and for the treatment of AA contained 2 mol% 5'-fluoro-2'-deoxyuridine dipalmitate (FUdR-dP; that is, 0.06 mol FudR-dP per 3 mol main lipid constituents) (synthesized as described previously [13]).

Lipids (and FUdR-dP or DiD) were dissolved in chloroform/methanol (9:1) and mixed. A lipid film was prepared through rotary evaporation under vacuum and dried under nitrogen. The lipids were hydrated with HN buffer (4-(2-hydroxyethyl)-1-piperazine ethanesulphonic acid (HEPES) and 135 mM NaCl) at pH 6.7. The resulting vesicles were sized by repeated extrusion through 100 nm polycarbonate filters. Particle size and size distribution were determined by dynamic laser light scattering with an Autosizer 4700 Spectrometer (Malvern Instruments, Malvern, Worcestershire, UK). Liposome preparations had a mean particle diameter ranging from 100 to 200 nm (polydispersity between 0.1 and 0.2). Typically, the mean liposomal diameter varied by less than 20% within any given experiment. The anti-CD134 or IgG2b isotype control mAbs were coupled to liposomes by a thiol-maleimide method described previously [13]. In brief, free thiol groups were introduced in the mAbs using the heterobifunctional reagent *N*-succinimidyl-*S*-acetylthioacetate (SATA; Sigma-Aldrich Chemie). Free SATA was separated from the derivatized mAbs by gel permeation chromatography, resulting in ATA-derivatized mAbs dissolved in HN buffer at pH 7.4. mAbs with reactive thiol groups, induced by deacetylating the ATA-protein, were incubated with liposomes at 4°C overnight at a ratio of 0.05 to 0.1 mg of mAbs per μmol lipid. *N*-ethylmaleimide (8 mM in HN buffer, pH 7.4) was added to cap unreacted thiol groups. Unconjugated mAbs were removed by gel-permeation chromatography or by centrifugation at 100,000 *g*. The liposomal protein content was determined as described previously [14]. Liposomes contained 25 to 125 μg of mAbs per μmol of lipid. Typically, the mAb content of the different liposome preparations within any given experiment varied by less than 20%.

### Liposome binding to CD4^+ ^T cells *in vivo*

On day 7 after Mt immunization, rats received saline or 5 μmol (lipid) DiD-labeled liposomes subcutaneously (s.c.) in each hind paw. After 30 min the rats were killed, and the PLN, ILN, and spleens were isolated. Single-cell suspensions were prepared as described above. Subsequently, cells were stained with anti-CD4-FITC, anti-CD134-biotin/streptavidin-PE and anti-TCR-αβ-PerCP, or with anti-CD45RA-FITC, anti-CD134-biotin/streptavidin-PE and anti-TCR-αβ-PerCP. Cell-associated fluorescence was measured by flow cytometry.

### Liposomal drug delivery to T cells *in vitro*

A2b T cells were activated *in vitro *to induce CD134 expression by stimulation overnight with 2.5 μg/ml Con A (Sigma-Aldrich Chemie) in the presence of 30 Gy-irradiated Lewis thymocytes as APC (ratio of T cells to APC = 1:25). Alternatively, a spleen cell suspension (at 2 × 10^5 ^cells/ml) was stimulated for 3 days with 2.5 μg/ml Con A to induce CD134 expression on splenic T cells. Next, viable cells were collected from the culture by Ficoll-Isopaque gradient centrifugation and transferred to round-bottomed 96-well plates at 10^5 ^cells per sample.

For studying anti-CD134 liposome binding to T cells *in vitro *by flow cytometry, A2b cells were incubated with 5 nmol (lipid) of anti-CD134 liposomes or IgG2b isotype control liposomes, or anti-CD134 mAb or IgG2b isotype control mAb for 30 min on ice. Cells were then washed and incubated on ice with FITC-conjugated goat anti-mouse immunoglobulin to label the cell-bound mAbs or liposomes. Finally, cell-associated fluorescence was measured. For analysis of the interaction of CD4^+ ^T cells and liposomes by confocal microscopy, activated A2b cells were incubated with 50 nmol of the different liposomal formulations in medium for 30 min on ice, washed and cultured in medium with 2% heat-inactivated normal rat serum at 37°C in 5% CO_2_. Activated spleen cells were incubated with 100 nmol of liposomes. At the indicated time points, cell-associated fluorescence was assessed.

For assessment of *in vitro *drug delivery by anti-CD134 liposomes, activated A2b cells were incubated at 37°C in 5% CO_2 _without or with 1 nmol (lipid) of the different liposomal formulations per well or with an equal concentration (100 nM) of free FUdR (Sigma-Aldrich Chemie) in 200 μl of medium without serum. After 30 min, cells were washed three times in medium and cultured for 48 hours in 200 μl of conditioned medium (medium supplemented with 10% heat-inactivated fetal calf serum (Bodinco, Alkmaar, The Netherlands), 10% culture supernatant of the EL-4 lymphoma (containing murine IL-2) and 1% non-essential amino acids (Invitrogen)). Finally, cells were pulsed for 18 to 20 hours with [^3^H]thymidine as described above, after which [^3^H]thymidine incorporation was measured. Results are expressed as the mean percentage of inhibition of proliferation of duplicate cultures relative to the incubation without liposomes (defined as 0%).

### Treatment of AA with liposomes and *ex vivo *proliferation assay of LN cells after liposomal treatment

AA was induced in Lewis rats as described above. Rats received 5 μmol of the different liposome formulations s.c. in each hind paw or HN buffer (see below) as a control on days 3 and 7 or on days 3, 7, and 10 after Mt immunization. Rats were followed for arthritis development as described above.

Proliferation of lymphocytes from liposome-treated animals was measured in quadruple cultures of 2 × 10^5 ^cells per well without additional APC. Cells were cultured in 96-well flat-bottomed plates in 200 μl of medium containing 2% heat-inactivated normal rat serum in the absence or presence of antigen (20 μg/ml Mt HSP60_176–190 _or 20 μg/ml OVA_232–339_) or Con A (2.5 μg/ml). After 72 hours, cells were pulsed for 18 to 20 hours with [^3^H]thymidine as described above, after which [^3^H]thymidine incorporation was measured.

### Statistical evaluation

The statistical significance of differences was evaluated with GraphPad Prism 3.02 (GraphPad Software, San Diego, California, USA). For statistical analysis of CD134 expression *in vivo *and liposomal drug delivery *in vitro*, a one-way analysis of variance with Dunnett's post-hoc test was used. For analysis of differences in the development of AA, a Mann-Whitney test was used for arthritis scores and an unpaired Student's *t*-test for body weight.

## Results

### Expression of CD134 on CD4^+ ^T cells during AA

To study the expression of CD134 and CD4 during AA, Lewis rats were immunized with Mt in adjuvant. The first signs of inflammation of the paw joints were observed between days 10 and 14, and the disease reached maximum severity at days 20 to 22. After this, inflammation of paw joints gradually decreased and resolved macroscopically at days 35 to 40. At several time points during AA development, the PLN (which drain the foot and ankle joints), the ILN (which drain the Mt immunization site), the spleen, and blood were isolated and examined by flow cytometry.

**Figure 1:**
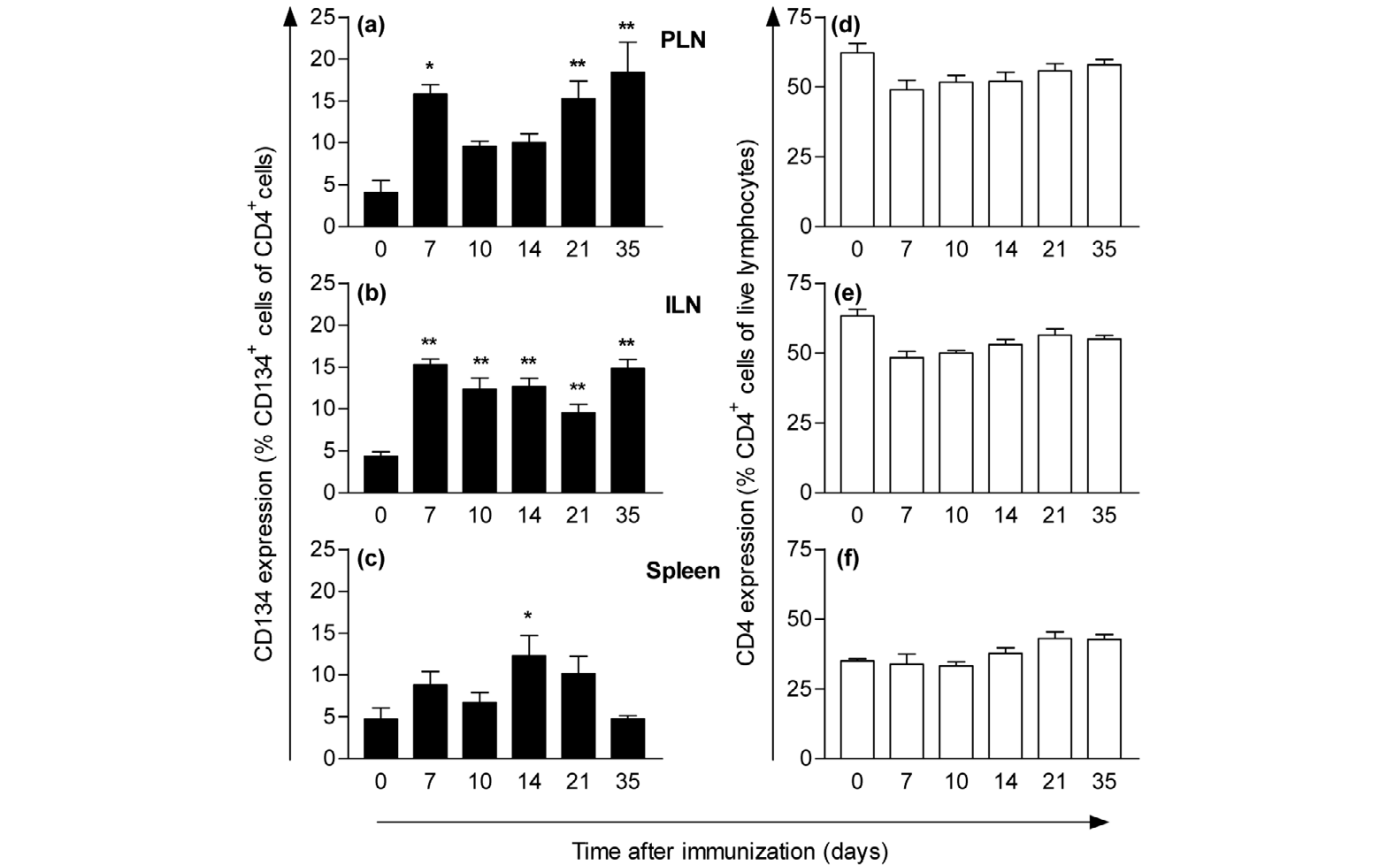
CD134 is differentially expressed on CD4^+ ^cells in secondary lymphoid organs during adjuvant arthritis. Popliteal lymph nodes **(a,d)**, inguinal lymph nodes **(b,e)**, and spleens **(c,f) **were isolated from Lewis rats before or during adjuvant arthritis (AA) development. Cell suspensions were stained for CD4 and CD134, and cell-associated fluorescence was analyzed by flow cytometry. Results for CD134 (black bars) are depicted as percentages of CD134^+ ^cells of the CD4^+ ^cell population and are expressed as means ± SEM (corrected for isotype control fluorescence). Results for CD4 (white bars) are shown as percentages of CD4^+ ^cells of the live lymphocytes population and are expressed as means ± SEM (live lymphocytes gated based on forward scatter (FSC) and side scatter (SSC) profiles). The data shown are derived from four independent experiments and represent *n *= 5 to 7 rats per group for *t *= 0, *n *= 2 rats per group for *t *= 7, *n *= 3 to 7 rats per group for *t *= 10, *n *= 5 to 8 rats per group for *t *= 14, *n *= 5 to 9 rats per group for *t *= 21, and *n *= 4 to 6 rats per group for *t *= 35. *P < 0.05 compared with *t *= 0, **P < 0.01 compared with *t *= 0.

Seven days after Mt immunization, before the clinical onset of AA, the percentage of CD134^+ ^T cells was increased both in the PLN and ILN in comparison with naive animals (day 0; Fig. 1a,b). In the ILN this percentage remained elevated throughout the active disease phase between days 10 and 30 (Fig. 1b). In the PLN a decrease in the percentage of CD134^+ ^T cells on days 10 and 14 was observed. On day 21 the percentage of CD134^+^CD4^+ ^cells was found to increase again (Fig. 1a). The total cell number in the PLN at day 7 (8.5 × 10^6 ^± 1.6 × 10^6^, mean ± SEM) and day 10 (8.3 × 10^6 ^± 2.7 × 10^6^) was comparable, as well as the percentage of CD4^+ ^cells (percentage of live lymphocytes; Fig. 1d). This indicated that the absolute number of CD134^+^CD4^+ ^T cells decreased during the interval from day 7 to day 10. In the spleen, the main increase in the percentage of CD134-expressing T cells was observed at about day 14 (Fig. 1c). In peripheral blood, no changes were detected in the percentage of CD134^+ ^cells during the onset of AA (data not shown). We did not observe a significant increase in the percentage of CD4^+ ^cells during AA in any of the organs tested (Fig. 1d–f). The data shown in Fig. 1 represent a compilation of four separate experiments, in which all rats were immunized on one day and flow cytometric analysis was performed on separate days. Another experiment in which rats were immunized on separate days (*n *= 4 rats per time point), and flow cytometric analysis was performed on one day, yielded similar results (data not shown).

### Specific responsiveness of CD134^+ ^T cells to the disease-associated epitope of Mt HSP60

**Figure 2:**
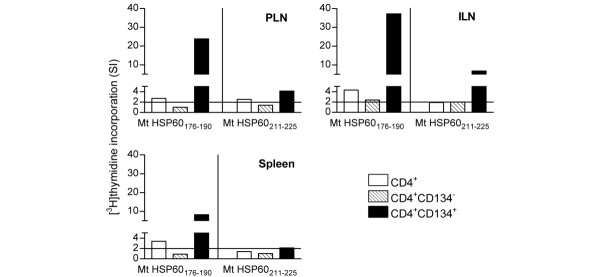
CD134+ T cells recognize the disease-associated mycobacterial epitope early in adjuvant arthritis. Popliteal lymph nodes, inguinal lymph nodes, and spleens were isolated from *n *= 13 rats at day 10 after immunization with *Mycobacterium tuberculosis *(Mt). The organs were pooled by organ type, and single-cell suspensions were stained for CD4 and CD134. The cells were sorted into CD4^+ ^(white bars), CD4^+^CD134^- ^(hatched bars), and CD4^+^CD134^+ ^(black bars) fractions. Proliferative responses to 20 μg/ml Mt HSP60_176–190 _(in which HSP60 stands for heat shock protein 60) were tested in a [^3^H]thymidine incorporation assay. As a control, the proliferation in response to 20 μg/ml Mt HSP60_211–225 _(not related to disease) was tested. Results are expressed as the mean SI of triplicate wells. The cut-off value for proliferation was set at SI = 2 (indicated by the horizontal line). Shown is one representative experiment of three.

Previously, it has been shown that a CD4^+ ^T cell clone (clone A2b [15]), derived from a Lewis rat after Mt immunization and capable of transferring arthritis to naive rats, recognized a T cell epitope present in the 176–190 region of Mt HSP60 (Mt HSP60_176–190_) [11]. To investigate whether CD134^+ ^T cells early in AA were potentially arthritogenic, we tested CD134^+^CD4^+ ^T cells isolated at days 7 and 10 after Mt immunization – that is, just before the onset of clinical disease – for their proliferative response to peptide Mt HSP60_176–190_. The results for day 10 are presented in Fig. 2. PLN-derived CD4^+ ^cells showed a low proliferative response to the disease-associated peptide (SI ≈ 3; Fig. 2). When the CD4^+ ^population was divided into CD134^+ ^and CD134^- ^fractions, the Mt HSP60_176–190 _response of the CD4^+ ^cells was entirely attributable to the CD134^+ ^cells, as these cells showed a high proliferative activity to Mt HSP60_176–190_, whereas this response was absent in the CD134^- ^population (SI < 2). Similar results were found for the CD4^+ ^subsets isolated from ILN and spleen (Fig. 2). The isolated CD134^+^CD4^+ ^cells also showed a response to another mycobacterial HSP60 epitope, peptide 211–225, which has been reported not to be related to AA [16]. However, this response was much lower than the Mt HSP60_176–190 _response (Fig. 2). Data obtained at day 7 (data not shown) and day 10 were similar. Thus, the CD134^+ ^T cell population found early in AA was enriched for activated auto-aggressive CD4^+ ^T cells, as shown by the specific response to the disease-associated epitope Mt HSP60_176–190_.

### Specific targeting to CD134^+ ^T cells in draining lymph nodes with anti-CD134 liposomes

**Figure 3:**
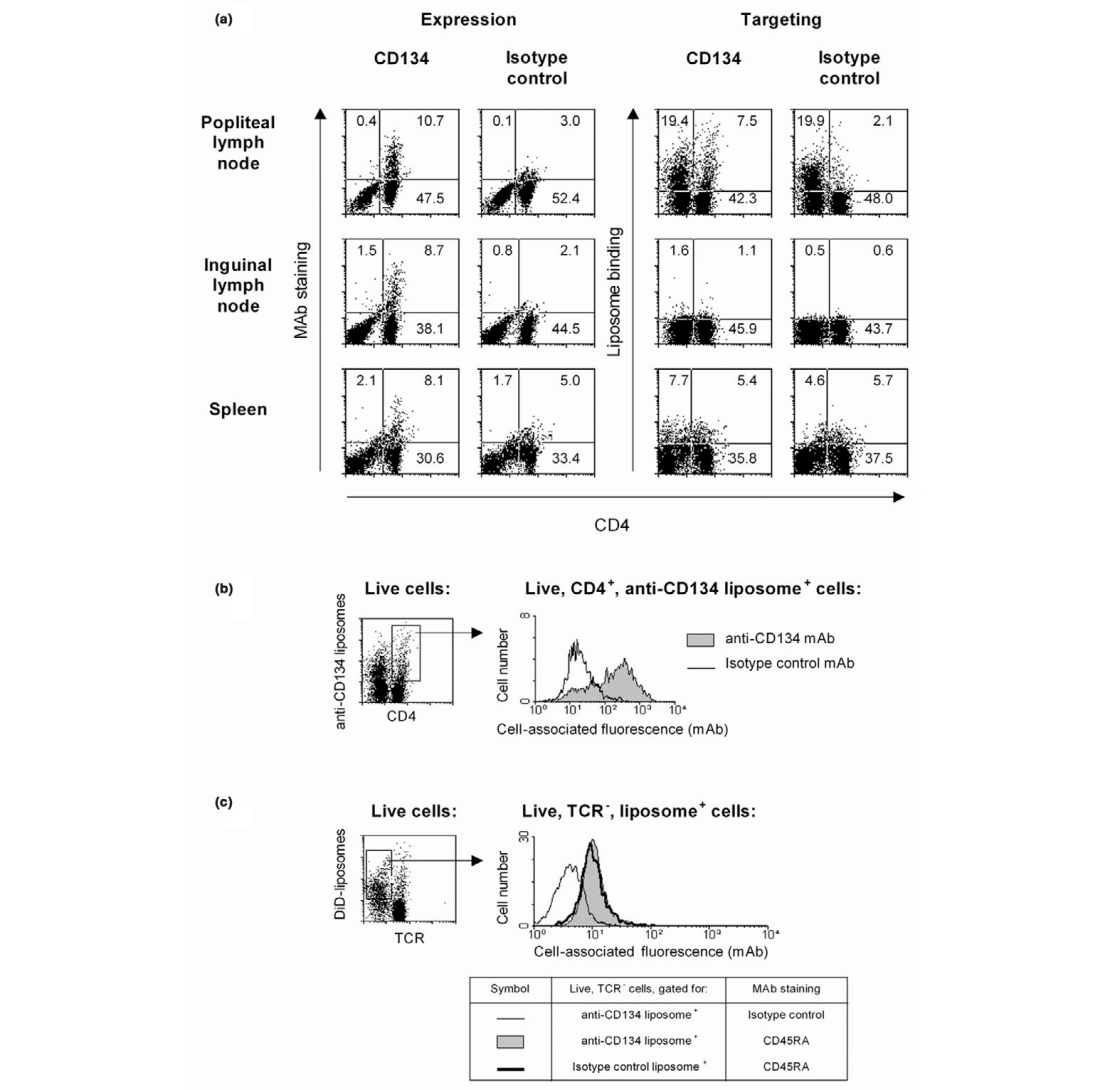
CD134^+ ^T cells in joint-draining lymph nodes are targeted by subcutaneous injection of anti-CD134 liposomes. On day 7 after immunization with *Mycobacterium tuberculosis *(Mt), rats were injected subcutaneously with fluorescent isotype control liposomes or anti-CD134 liposomes. Rats were killed 30 min later, and popliteal lymph nodes (PLN), inguinal lymph nodes, and spleens were isolated. **(a) **Cells were stained for CD4 and T-cell antigen receptor (TCR)-αβ and cell-associated fluorescence was analyzed by flow cytometry. Dot plots show cell-associated fluorescence due to *in vitro *monoclonal antibody (mAb) staining (left panels) or *in vivo *liposome binding (right panels). Cells were gated for live TCR-αβ^+ ^CD4^+ ^cells. The numbers in the dot plots indicate the percentage of cells above the cut-off line, which was set by using non-stained cells from sham-injected animals. Three rats were analyzed per group; representative stainings of one rat per group were selected and are shown here. **(b) **PLN cells of anti-CD134 liposome-injected rats were stained with anti-CD4 and anti-CD134 or its isotype control. Cells were gated for live CD4^+^liposome^+ ^cells. Histograms show cell-associated fluorescence due to the binding of anti-CD134 (filled) or isotype control mAb (open). Representative stainings of one rat of three are shown. **(c) **PLN cells were stained with anti-TCR-αβ and anti-CD45RA (rat B cells). Cells were gated for live, TCR-αβ^-^, and liposome^+ ^cells. Histograms show cell-associated fluorescence due to *ex vivo *CD45RA (filled histogram) or isotype control mAb staining (thin line) on anti-CD134 liposome^+ ^cells, or CD45RA (thick line) mAb staining on isotype control liposome^+ ^cells. Representative stainings of one rat of three are shown.

For delivery of modulating compounds to the potentially arthritogenic CD134^+ ^T cells, we selected a mAb-targeted liposomal system. To investigate whether the CD134^+ ^CD4^+ ^T cells in the draining LN could be targeted *in vivo*, fluorescent anti-CD134 liposomes were injected s.c. in the hind paws of rats on day 7 after Mt immunization. After 30 min, rats were killed, and the T cells in the joint-draining PLN, the immunization site-draining ILN and spleen were studied for CD134 expression and for the presence of cell-bound liposomes by flow cytometry. In the PLN, 10.7% of the cells were found to be both CD4^+ ^and CD134^+^, whereas 7.5% of the cells had bound anti-CD134 liposomes and were CD4^+ ^(Fig. 3a). Competitive counterstaining of liposome^+^CD4^+ ^cells with anti-CD134 mAbs showed that virtually all these cells were indeed CD134^+ ^(Fig. 3b). This implies that the vast majority of CD134^+ ^T cells in the PLN was targeted. In addition, also CD4^- ^cells were targeted in the PLN (Fig. 3a). In this case, however, binding of both anti-CD134^- ^and isotype control liposomes was comparable and these cells were determined to be CD45RA^+ ^B cells (Fig. 3c). Because B cells do not express CD134, the anti-CD134 binding could not be due to CD134 binding. The similar staining pattern of isotype control liposomes and anti-CD134 liposomes on B cells suggested that this binding was due to Fc-mediated binding (because whole mAb was used to coat liposomes). In the ILN or the spleen virtually no CD134^+ ^CD4^+ ^T cells were targeted by anti-CD134 liposomes administered s.c. in the paw (Fig. 3a).

### Drug delivery to CD134^+ ^T cells *in vitro *using anti-CD134 liposomes containing the dipalmitate-anchored cytostatic agent FUdR

**Figure 4:**
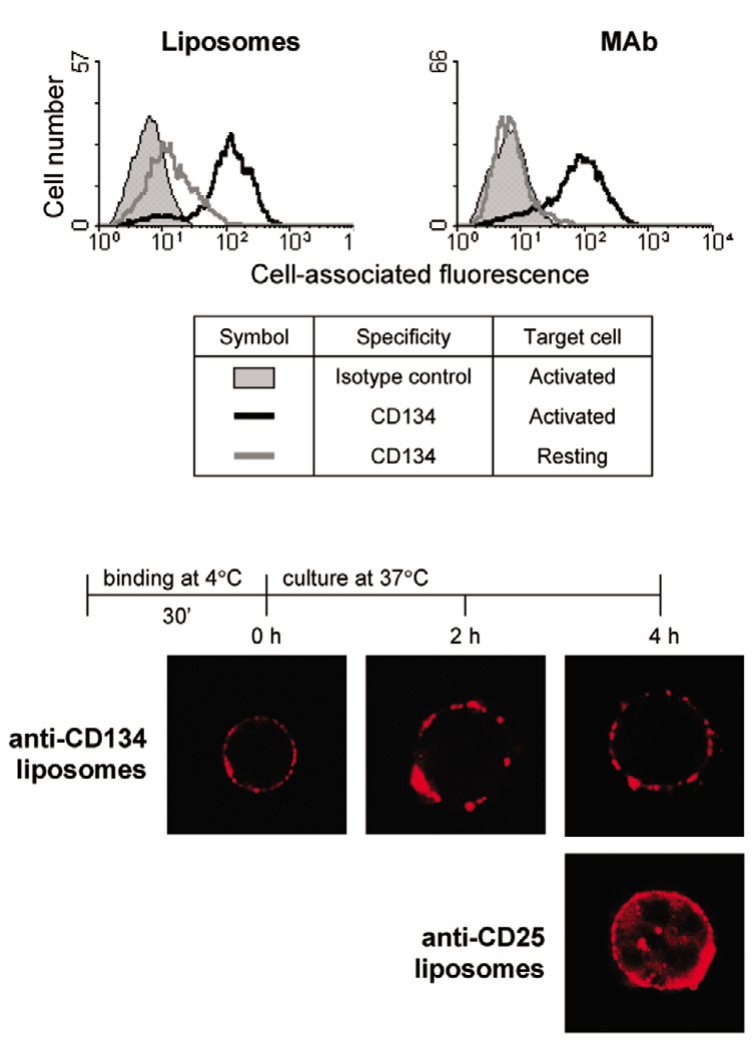
Anti-CD134-mediated targeting does not lead to liposome internalization by activated CD4^+ ^T cells *in vitro*. A2b T cells were cultured with antigen-presenting cells and Con A to induce CD134 expression; CD25 is expressed constitutively on these cells. **(a) **Viable T cells were incubated with isotype control liposomes (filled histogram) or with anti-CD134 liposomes (black line). As a control, the binding of anti-CD134 liposomes or anti-CD134 monoclonal antibodies to resting T cells was assessed (gray lines). Cell-associated fluorescence was analyzed by flow cytometry, with live cells gated on the basis of forward scatter (FSC) and side scatter (SSC) profiles. One representative experiment of three is shown. **(b) **Viable T cells were incubated for 30 min with anti-CD134 liposomes on ice. After the removal of non-bound liposomes by washing, cells were cultured subsequently at 37°C. Samples were taken at the indicated time points and analyzed for the cellular localization of the liposomal fluorescence with the use of confocal microscopy. A representative cell from each time point is shown. As a positive control for cellular internalization of liposomes, cells incubated with anti-CD25 liposomes are shown. One of two experiments, yielding similar results, is shown.

The fate of anti-CD134 liposomes after binding to activated CD4^+ ^T cells was studied *in vitro *by incubation of activated T cells of clone A2b with anti-CD134 liposomes. Activated CD134^+ ^A2b T cells were shown to specifically bind anti-CD134 liposomes (Fig. 4a). Interestingly, although resting A2b cells seemed CD134^- ^after conventional mAb staining, anti-CD134 liposomes did bind to these cells to a small extent.

Using confocal microscopy, anti-CD134 liposomes were shown to bind specifically to activated CD4^+ ^T cells in a diffuse pattern; that is, spread out over the plasma membrane (Fig. 4b). When cells that had bound liposomes were incubated at 37°C, the staining pattern of anti-CD134 liposomes changed from diffuse to a more focal pattern after 2 hours of culture at 37°C (Fig. 4b, 2 hours). However, no internalization of anti-CD134 liposomes was observed at any of the time points evaluated. This was also observed with Con A-activated splenic T cells (data not shown). As a positive control for liposome internalization, liposomes targeting CD25, the α-subunit of the IL-2 receptor, which is also expressed on activated CD4^+ ^T cells, were used (Fig. 4b, 4 hours). Furthermore, activated CD4^+ ^T cells were able to internalize anti-CD134 mAbs (and anti-CD25 mAbs) within 2 hours of binding (data not shown). This indicated that although the CD134 receptor itself was internalized, cell-bound anti-CD134 liposomes were not internalized by the targeted T cells.

Our finding that anti-CD134 liposomes were not internalized by the target T cells had major implications for the strategy of drug delivery. We decided to use the mechanism of lipid-coupled drug transfer between membranes to achieve intracellular drug delivery. The lipid-derivatized cytostatic agent FUdR-dP was used as a model drug [13]. Activated, CD134^+ ^rat T cells of clone A2b were found to be very sensitive to free FUdR (90% growth inhibition with 100 nM FUdR) when FUdR was continuously present during culture for 48 hours. However, when the cells were incubated for only 30 min with free FUdR and then washed to remove extracellular FUdR, no significant growth inhibition was detected during subsequent culture (Table 1). When the equivalent amount of FUdR was present in 1 nmol anti-CD134 liposomes (anti-CD134-FUdR-dP liposomes), which were incubated for 30 min with the cells, the proliferation of activated A2b cells was inhibited by more than 30% (Table 1). Incubation of activated T cells with anti-CD134 liposomes without FUdR-dP or with non-targeted FUdR-dP liposomes did not significantly affect the proliferation of the cells (*P *> 0.05; Table 1).

### Modulation of AA by treatment with drug-containing anti-CD134 liposomes

**Figure 5:**
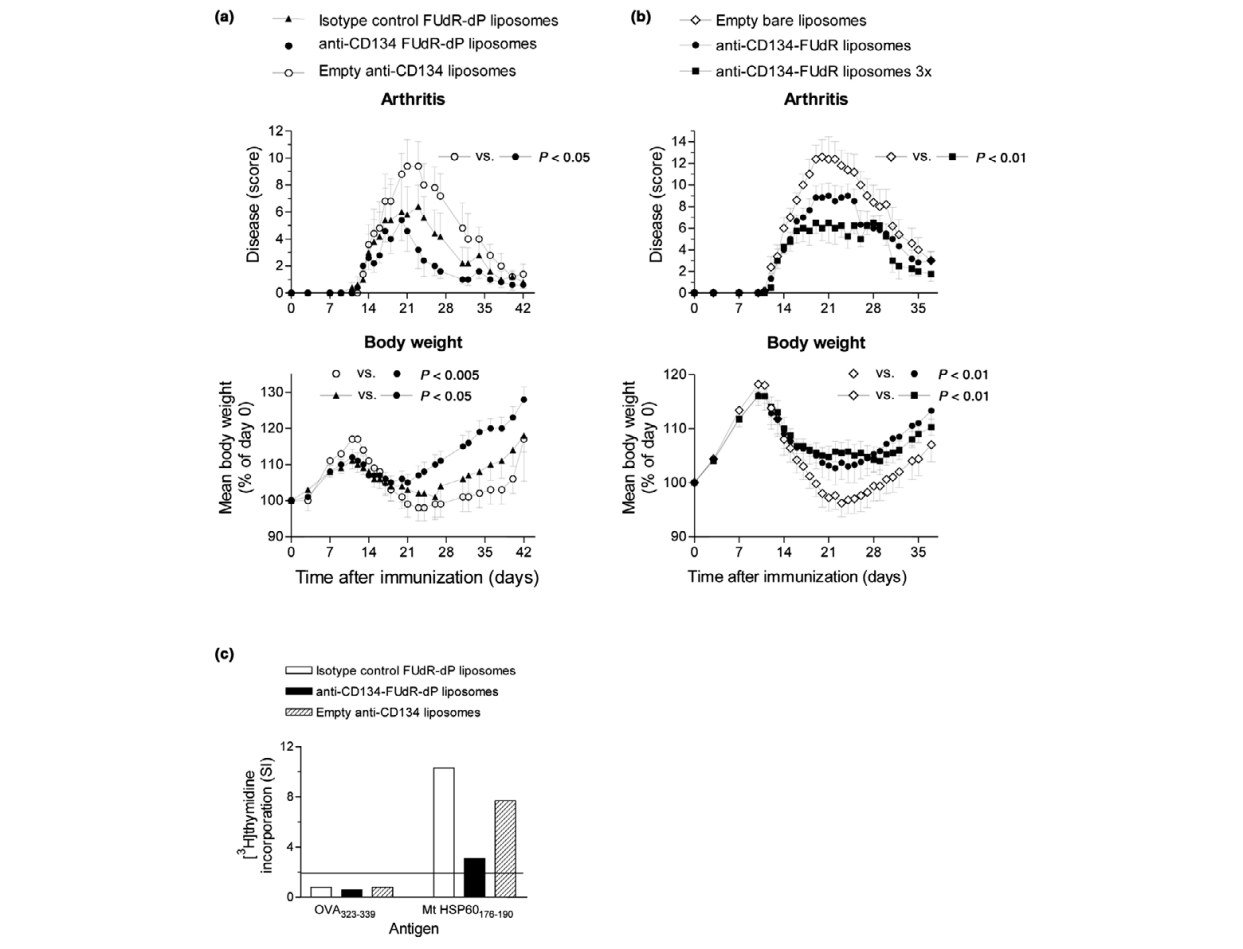
Adjuvant arthritis is modulated by treatment with anti-CD134 5'-fluoro-2'-deoxyuridine dipalmitate (FUdR-dP) liposomes. **(a) **Rats were immunized with *Mycobacterium tuberculosis *(Mt) to induce arthritis. On days 3 and 7, rats received isotype control FUdR-dP liposomes (filled triangles), anti-CD134-FUdR-dP liposomes (filled circles), or empty anti-CD134 liposomes (open circles) subcutaneously (s.c.) in both hind paws. **(b) **Alternatively, after immunization with Mt, on days 3 and 7 rats received anti-CD134-FUdR-dP liposomes (filled circles) or empty bare liposomes (open diamonds), or on days 3, 7, and 10 anti-CD134-FUdR-dP liposomes (filled squares), s.c. in both hind paws. Rats were followed for the development of clinical disease and body weight until the disease resolved spontaneously (day 37 to 42). Results are expressed as the arthritis score and the mean body weight (percentage of day 0) per group of *n *= 5 rats and are presented as means ± SEM. Statistical differences are indicated in the plots. **(c) **On day 42, rats shown in **(a) **were killed; popliteal lymph node cells were isolated and pooled from each treatment group. Cells from isotype control FUdR-dP liposome-treated rats (white bars), anti-CD134-FUdR-dP liposome-treated rats (black bars), and empty anti-CD134 liposome-treated rats (hatched bars) were tested for their proliferative response to 20 μg/ml Mt HSP60_176–190 _peptide (in which HSP60 stands for heat shock protein 60) in a [^3^H]thymidine incorporation assay. The proliferation to 20 μg/ml peptide OVA_323–339 _is shown as a negative control. Results are expressed as the mean SI for quadruple wells. The cut-off value for proliferation was set at SI 2 (indicated by line).

Next, we investigated whether local targeting to CD134^+ ^T cells in the joint-draining PLN would affect the course of actively induced arthritis in the AA model. Rats were injected with the different liposomal formulations on days 3 and 7 after Mt immunization and were followed for arthritis development. Injection of anti-CD134-FUdR-dP liposomes resulted in less severe disease development than in rats injected with anti-CD134 liposomes without FUdR-dP (Fig. 5a). This effect was increased by the administration of anti-CD134-FudR-dP liposomes on three occasions (Fig. 5b). The improved well-being of the anti-CD134-FUdR-dP liposome-treated rats was also reflected in a faster recovery of weight (Fig. 5a). The isotype control FUdR-dP liposomes also seemed to affect the progression of AA, although anti-CD134-FUdR-dP liposomes were more effective. No difference in AA scores was found between rats treated with empty anti-CD134 liposomes and rats treated with empty, bare liposomes (data not shown).

The modulation of the course of AA after treatment with anti-CD134-FUdR-dP liposomes was correlated with a decreased proliferative response to Mt HSP60_176–190 _of joint-draining PLN cells isolated at day 42 (Fig. 5c). This is indicative of successful targeting and deletion of Mt HSP60_176–190_-reactive, CD134^+ ^T cells *in vivo*.

## Discussion

In the present study we investigated whether CD134 can be used as a (transient) marker for targeting auto-aggressive CD4^+ ^T cells in actively induced experimental arthritis. Before the onset of clinical arthritis, an elevated percentage of CD134^+ ^CD4^+ ^T cells was found in the PLN, which drain the foot and ankle joints, and in the ILN, which drain the Mt immunization site. In the ILN, this percentage remained elevated throughout the active disease phase, indicating a continuous activation of T cells, probably because of the presence of an Mt depot at the base of the tail. However, in the PLN the percentage and absolute number of CD134^+ ^T cells were decreased at days 10 and 14 after immunization in comparison with the initial elevation on day 7. In this arthritis model, the first signs of clinical disease become manifest between days 10 and 14, and at about this time T cells start to infiltrate the joints [17]. The present data therefore suggest that early in AA, CD134^+ ^T cells, after activation in the PLN, can migrate to the joints, where they subsequently become involved in joint inflammation. The second increase in the percentage of CD134-expressing T cells in the PLN on days 21 and 35 could reflect the recirculation or generation of activated auto-aggressive T cells, or the emergence of an activated regulatory population.

The presence of arthritogenic cells in the CD134^+ ^subset of CD4^+ ^T cells isolated from pre-arthritic rats was deduced from the high proliferative response to the Mt HSP60_176–190 _peptide, which was previously linked to the induction of AA [11]. However, the CD134^+ ^T cell subset also includes activated (auto-aggressive) T cells with a different specificity. The low but evident response of isolated CD4^+^CD134^+ ^cells to Mt HSP60_211–225_, which was reported not to be related to AA [16], indeed indicated that a part of the CD134^+ ^T cells was not activated in relation to clinical disease but responded to other epitopes present in the immunization mix. This underlines the fact that, although CD134 may be used to select for activated pathogenic CD4^+ ^T cells in an autoimmune setting, this molecule is primarily a marker for activated CD4^+ ^T cells in general [18-20]. Nevertheless, by using CD134 as marker for targeting, we expected to affect all recently activated auto-aggressive CD4^+ ^T cells present at the time of targeting, including arthritogenic T cells with a different specificity from that of Mt HSP60_176–190_. The preferential expression of CD134 on synovial fluid CD4^+ ^T cells from patients with rheumatoid arthritis, as has been demonstrated by others [6,7,21], indicates that also in humans auto-reactive T cells might be (transiently) marked by CD134. Because CD134 ligand expression has been demonstrated both on vascular endothelial cells [22,23] and in synovial tissue of rheumatoid arthritis patients [21], the recruitment and *in situ *restimulation of activated T cells through CD134 possibly contributes to the inflammatory process in arthritis. Indeed, in a mouse collagen-induced arthritis model, treatment with a mAb blocking anti-CD134 ligand did inhibit disease development [21].

To explore the possibility for modulating auto-aggressive T cells in arthritis, we examined the potential of drug targeting directly to CD134^+ ^T cells in AA by using liposomes as drug carriers. To study the ability of anti-CD134 liposomes to reach the potentially auto-reactive CD134^+ ^T cells *in vivo*, the active disease model was employed, because this would allow targeting of the target T cells during priming *in situ*; that is, in the secondary lymphoid organs. When anti-CD134 liposomes were injected s.c. in the hind paws, the majority of the CD4^+^CD134^+ ^T cells in the joint-draining PLN could indeed be targeted. The non-T cells in the PLN that were found to bind both anti-CD134 liposomes and isotype control liposomes were determined as being B cells that most probably bound the liposomes in a Fc-mediated fashion.

Activated CD134^+ ^T cells targeted by anti-CD134 liposomes *in vitro *did not internalize the cell-bound liposomes. This lack of internalization determined the strategy of drug delivery. We here employed the mechanism of lipid-coupled drug transfer between membranes to achieve intracellular drug delivery. When anti-CD134 liposomes carried the lipid-coupled cytostatic agent FUdR as a model drug, a 30% inhibition of proliferation of activated CD134^+ ^T cells was observed *in vitro*. This inhibitory effect on the proliferation of CD134^+ ^T cells *in vitro *was correlated with a moderate suppression of AA in rats treated with anti-CD134-FUdR-dP liposomes. The effect of these liposomes on AA development was supported by a downregulation of the disease-associated Mt HSP60_176–190 _response in the PLN of anti-CD134-FUdR-dP liposome-treated animals.

The effect of isotype control-FUdR-dP on clinical disease might be due to their association with B cells *in vivo*, probably through binding to Fc receptors [24]. Although B cells have not been described as having a crucial role in the development of AA [25], contrary to collagen-induced arthritis in mice, for example [26], these cells can function as APC and as such may affect the response of auto-aggressive T cells *in vivo*. Optimizing the therapeutic entity, for example by coupling anti-CD134 Fab fragments to the liposomal carrier instead of the entire anti-CD134 antibody, would circumvent B cell targeting.

It has recently been shown that CD4^+^CD25^+ ^regulatory T cells involved in the control of autoimmunity [27] can express CD134 [28-30]. Although CD134^+ ^regulatory T cells may go through a proliferative phase *in vivo *[31,32], in general these cells display a hypoproliferative phenotype *in vitro *as well as *in vivo *[33]. Because cytostatic agents act primarily on proliferating cells, it is possible that by employing FUdR-dP-containing anti-CD134 liposomes we largely preserved this regulatory T cell subset.

## Conclusion

We show here that CD134 can be used as a marker for recently activated CD4^+ ^T cells with auto-aggressive potential in arthritis, and that anti-CD134 liposomes can be used to target drugs directly to these T cells. Thus, anti-CD134 liposomes represent an attractive method for the development of therapies aiming at the modulation of auto-aggressive T cells for intervention in autoimmune diseases.

## Abbreviations

AA = adjuvant arthritis; APC = antigen-presenting cells; Con A = concanavalin A; DiD = 1,1'-dioctadecyl-3,3,3',3'-tetramethylindodicarbocyanine, 4-chlorobenzenesulfonate salt; FITC = fluorescein isothiocyanate; FUdR(-dP) = 5'-fluoro-2'-deoxyuridine (dipalmitate); HSP = heat shock protein; ILN = inguinal lymph nodes; mAb = monoclonal antibody; Mt = *Mycobacterium tuberculosis*; PBS = phosphate-buffered saline; PE = phycoerythrin; PerCP = peridinin chlorophyll protein; PLN = popliteal lymph nodes; s.c. = subcutaneously; SI = stimulation index; TCR = T-cell antigen receptor.

## Competing interests

The author(s) declare that they have no competing interests.

## Authors' contributions

EB participated in designing performing the experiments and prepared the manuscript. GK participated in, and supervised, the liposome preparations. GS participated in the design and coordination of the study, and in the interpretion of the results. JWH carried out immunological experiments and participated in the interpretation of the results. WVE participated in the design of the study and in its coordination. LE participated in the experiments, statistical analysis and interpretation of the results. MW supervised the design and execution of the study, and drafted the manuscript. All authors read and approved the final manuscript.

## Figures and Tables

**Table 1:** Anti-CD134 5'-fluoro-2'-deoxyuridine dipalmitate liposomes inhibit the proliferation of CD134^+ ^T cells *in vitro*

Drug	Drug carrier	Targeting moiety	Inhibition of proliferation (%)
None	None	None	0.0 ± 8.2
FUdR	None	Anti-CD134	8.9 ± 8.7
FUdR-dP	Liposomes	None	6.3 ± 0.7
None	Liposomes	Anti-CD134	0.6 ± 1.1
FUdR-dP	Liposomes	Anti-CD134	31.0 ± 2.3*

Con A-activated CD134^+ ^T cells of clone A2b were incubated for 30 min without ('none' under the heading 'drug') or with either free 5'-fluoro-2'-deoxyuridine (FUdR), 5'-fluoro-2'-deoxyuridine dipalmitate (FUdR-dP) liposomes, anti-CD134 liposomes, or anti-CD134-FUdR-dP liposomes. To each well was added 100 nM FUdR or 1 nmol of liposomal lipid (for FUdR-dP liposomes this equals 100 nM FUdR). Subsequently, cells were washed and cultured for 48 hours, followed by [^3^H]thymidine incorporation as a measure of proliferation. Results are expressed as the mean percentage of inhibition of proliferation relative to the incubation without liposomes (defined as 0%; mean [^3^H]thymidine incorporation of 53,330 c.p.m.). Results in the last column are means ± SEM. One representative experiment of three is shown. *P < 0.001 compared with incubation without liposomes.
